# Immunological Biomarkers of Fatal COVID-19: A Study of 868 Patients

**DOI:** 10.3389/fimmu.2021.659018

**Published:** 2021-05-03

**Authors:** Esperanza Martín-Sánchez, Juan José Garcés, Catarina Maia, Susana Inogés, Ascensión López-Díaz de Cerio, Francisco Carmona-Torre, Marta Marin-Oto, Félix Alegre, Elvira Molano, Mirian Fernandez-Alonso, Cristina Perez, Cirino Botta, Aintzane Zabaleta, Ana Belen Alcaide, Manuel F. Landecho, Marta Rua, Teresa Pérez-Warnisher, Laura Blanco, Sarai Sarvide, Amaia Vilas-Zornoza, Diego Alignani, Cristina Moreno, Iñigo Pineda, Miguel Sogbe, Josepmaria Argemi, Bruno Paiva, José Ramón Yuste

**Affiliations:** ^1^ Hematology Department, Clínica Universidad de Navarra, Pamplona, Spain; ^2^ Hemato-Oncology Department, Centro de Investigación Médica Aplicada (CIMA), Pamplona, Spain; ^3^ Hemato-Oncology Department, Instituto de Investigación Sanitaria de Navarra (IdiSNA), Pamplona, Spain; ^4^ Centro de Investigación Biomédica en Red, Pamplona, Spain; ^5^ Immunology and Immunotherapy Department, Clínica Universidad de Navarra, Pamplona, Spain; ^6^ Hematology Service and Cell Therapy Area, Clínica Universidad de Navarra, Pamplona, Spain; ^7^ Internal Medicine Department, Clínica Universidad de Navarra, Pamplona, Spain; ^8^ Division of Infectious Diseases, Clínica Universidad de Navarra, Pamplona, Spain; ^9^ Immune and Infectious Inflammatory Diseases Research, Instituto de Investigación Sanitaria de Navarra (IdiSNA), Pamplona, Spain; ^10^ Neumology Department, Clínica Universidad de Navarra, Pamplona, Spain; ^11^ Internal Medicine Department, Clínica Universidad de Navarra, Madrid, Spain; ^12^ Microbiology Department, Clínica Universidad de Navarra, Pamplona, Spain; ^13^ Hematology Department, Hospital “Annunziata”, Cosenza, Italy; ^14^ Neumology Department, Clínica Universidad de Navarra, Madrid, Spain

**Keywords:** COVID-19, SARS-CoV-2, flow cytometry, lymphopenia, outcome, survival, biomarkers

## Abstract

Information on the immunopathobiology of coronavirus disease 2019 (COVID-19) is rapidly increasing; however, there remains a need to identify immune features predictive of fatal outcome. This large-scale study characterized immune responses to severe acute respiratory syndrome coronavirus-2 (SARS-CoV-2) infection using multidimensional flow cytometry, with the aim of identifying high-risk immune biomarkers. Holistic and unbiased analyses of 17 immune cell-types were conducted on 1,075 peripheral blood samples obtained from 868 COVID-19 patients and on samples from 24 patients presenting with non-SARS-CoV-2 infections and 36 healthy donors. Immune profiles of COVID-19 patients were significantly different from those of age-matched healthy donors but generally similar to those of patients with non-SARS-CoV-2 infections. Unsupervised clustering analysis revealed three immunotypes during SARS-CoV-2 infection; immunotype 1 (14% of patients) was characterized by significantly lower percentages of all immune cell-types except neutrophils and circulating plasma cells, and was significantly associated with severe disease. Reduced B-cell percentage was most strongly associated with risk of death. On multivariate analysis incorporating age and comorbidities, B-cell and non-classical monocyte percentages were independent prognostic factors for survival in training (n=513) and validation (n=355) cohorts. Therefore, reduced percentages of B-cells and non-classical monocytes are high-risk immune biomarkers for risk-stratification of COVID-19 patients.

## Introduction

Severe acute respiratory syndrome coronavirus-2 (SARS-CoV-2) infections have resulted in 132 million confirmed cases of coronavirus disease 2019 (COVID-19), including almost 3 million deaths, worldwide (https://covid19.who.int/). Age, pre-existing medical conditions, male gender, and immune system hyperactivation are associated with a higher risk of severe disease (1–5). Patients with severe COVID-19 have abnormally high levels of pro-inflammatory cytokines, leukocytosis, and lymphopenia, although it is unknown whether these immune perturbations are associated with specific comorbidities or are independent drivers of COVID-19 severity (1). Furthermore, general inflammation increases with age, and this could either inhibit immunity to infections or initiate an inflammatory cascade, amplifying the excessive inflammation that occurs in response to pathogens (6). Thus, dissecting dysfunctional immune signatures in the context of age and comorbidities could be of paramount importance for optimal risk-stratification of COVID-19 patients and the design of tailored treatment strategies (7).

An increasing number of studies suggest that reduced innate antiviral defenses, coupled with heightened inflammation, are defining features of severe COVID-19 (8, 9). These observations using single-cell RNA-sequencing or mass cytometry have come from deep immune profiling of either nasopharyngeal, bronchial, and post-mortem lung samples or peripheral blood (PB) (10) from relatively small numbers of patients (8, 9, 11–18). Larger-scale analyses using flow cytometry have shown COVID-19 patients to have reduced numbers of CD4 and CD8 T cells, especially patients aged ≥60 years and/or those requiring intensive care unit (ICU) admission (1, 19–23). The importance of myeloid cells in severe COVID-19 has also been determined (16, 18, 24). Namely, an excessive inflammatory response to SARS-CoV-2 is a major cause of disease severity and death, and is associated with a profound lymphopenia, neutrophil activation, immune cell infiltration in several tissues, altered monocyte activation, and high levels of circulating cytokines (25, 26). Conversely, a loss of function in myeloid cells has also been described to mirror an immune pathological status progressing from immune paralysis to “immune silence”, which is associated with higher susceptibility to fatal COVID-19 (26). These data highlight the relevance of immune-monitoring for identifying patients who may become critically ill. However, they fall short of providing a comprehensive immunophenotypic atlas of COVID-19, probably due to limited data from large patient cohorts on the relative distribution of innate and adaptive immune cell-types, including pro-inflammatory granulocytic cells, in whole PB samples (10, 21–23, 27–29). Furthermore, a considerable number of immune features associated with SARS-CoV-2 infection are not being routinely used for risk-stratification of COVID-19 patients.

We hypothesized that a comprehensive analysis of immune responses in a large cohort of patients would accelerate our understanding of the immunopathobiology of COVID-19 and potentially identify immune biomarkers for risk-stratification that could be complementary to other well-known prognostic factors.

## Material and Methods

### Patients and Subjects

Between March and May 2020, 537 consecutive patients aged ≥18 years with clinical symptoms suggestive of COVID-19 were admitted to the Clinica Universidad de Navarra. Of these, 513 had a positive SARS-CoV-2 PCR test and/or presence of SARS-CoV-2-specific antibodies, and represent the exploratory series of COVID-19 patients investigated in this study. The remaining 24 patients had a negative SARS-CoV-2 PCR test and/or no SARS-CoV-2-specific antibodies, and were diagnosed instead with pneumonia (n=16), respiratory tract infections (n=4), soft tissue infection (n=1), infectious mononucleosis (n=1), urinary tract infection (n=1), and sepsis after infection (n=1). Between June 2020 and February 2021, another 355 COVID-19 patients aged ≥18 years were admitted to the Clinica Universidad de Navarra and represent the validation series.

Patients with COVID-19 were staged according to the clinical risk score of Liang et al. (30). General ward admission criteria included characteristic radiographic findings and/or shortness of breath defined as tachypnea and/or low oxygen pulse in absence of alternative diagnosis. Criteria for ICU admission included low oxygen pulse despite supplementary oxygen with non-rebreather mask, sepsis per Sequential Organ Failure Assessment (SOFA) criteria and/or requiring mechanical ventilation, shock requiring vasopressor drugs, and unexplained confusion. All patients received standard supportive care, including low-molecular-weight heparin, statins, and supplementary oxygen on demand. Patients with severe COVID-19, with oxygen saturation of ≤90%, additionally received corticosteroids, and critically ill patients received further tocilizumab. Antiviral therapy with hydroxychloroquine/azythromycin or ritonavir/lopinavir was administered depending on disease severity.

This study also included 36 healthy donors (HD) to provide an age-matched reference cohort for comparison of immune profiles. HDs had no prior diagnosis of or recent symptoms consistent with COVID-19, and had a negative SARS-CoV-2 PCR test at the time of sample collection.

### Study Design

PB samples were obtained for immune profiling from the 868 COVID-19 patients and the 24 patients with other infections at the time of admission. Additionally, subsequent longitudinal samples were collected over time (n=207) from 167 COVID-19 patients (**Supplemental Table 1**). The aim was to determine whether COVID-19 outcome was associated solely with immune status at presentation or if different immune response trajectories over time were associated with different outcomes. Relative changes in immune cell-type levels from presentation through subsequent PB sample collection time points were evaluated according to COVID-19 outcome. PB samples were also collected from the 36 HDs. Identical immunophenotyping was performed using multidimensional flow cytometry (MFC) on all 1,135 samples to characterize immune profiles in COVID-19 patients and to compare these with immune profiles in response to other pathogens and HDs. Moreover, a deep analysis of myeloid and T and B cells was carried out by transcriptomics and high-resolution flow cytometry, respectively, in a subset of 14 COVID-19 patients and 4 HDs.

The 36 HDs were segmented into four age groups to provide age-matched reference immune profiles for young adults (aged 18–30 years, n=8), middle-age adults (aged 31–55 years, n=8), elderly but more likely fit subjects (aged 56–70 years, n=11), and elderly and more likely unfit subjects (aged >70 years, n=9). COVID-19 patients were segmented into the same four age groups and immune profiles compared *versus* the respective HDs to determine variations in cell-type proportions.

The Clinica Universidad de Navarra Ethics Committee approved the protocol and informed consent forms, which patients were required to sign prior to enrollment. The study was conducted per the ethical principles of the Declaration of Helsinki.

### MFC Immunophenotyping and Automated Clustering of PB Immune Cells

EDTA anti-coagulated PB samples were stained with the 8-color combination of the monoclonal antibodies CD3-V450, CD45-V500, CD20-FITC, CD16-PE, CD4-PerCPCy5.5, CD19-PECy7, CD56-APC, and CD8-APCH7 (**Supplemental Table 2**), lysed for 30 min, and measured directly – without centrifugation and washing steps to minimize risk of infection – in a FACSCanto II flow cytometer (Beckton Dickinson Biosciences [BD], San Jose, CA, USA) using FACSDiva 6.1 software (BD). In a subset of 14 COVID-19 patients and 4 HDs, PB T and B cells were characterized using EuroFlow panels for primary immunodeficiencies (31).

**Figure 1:**
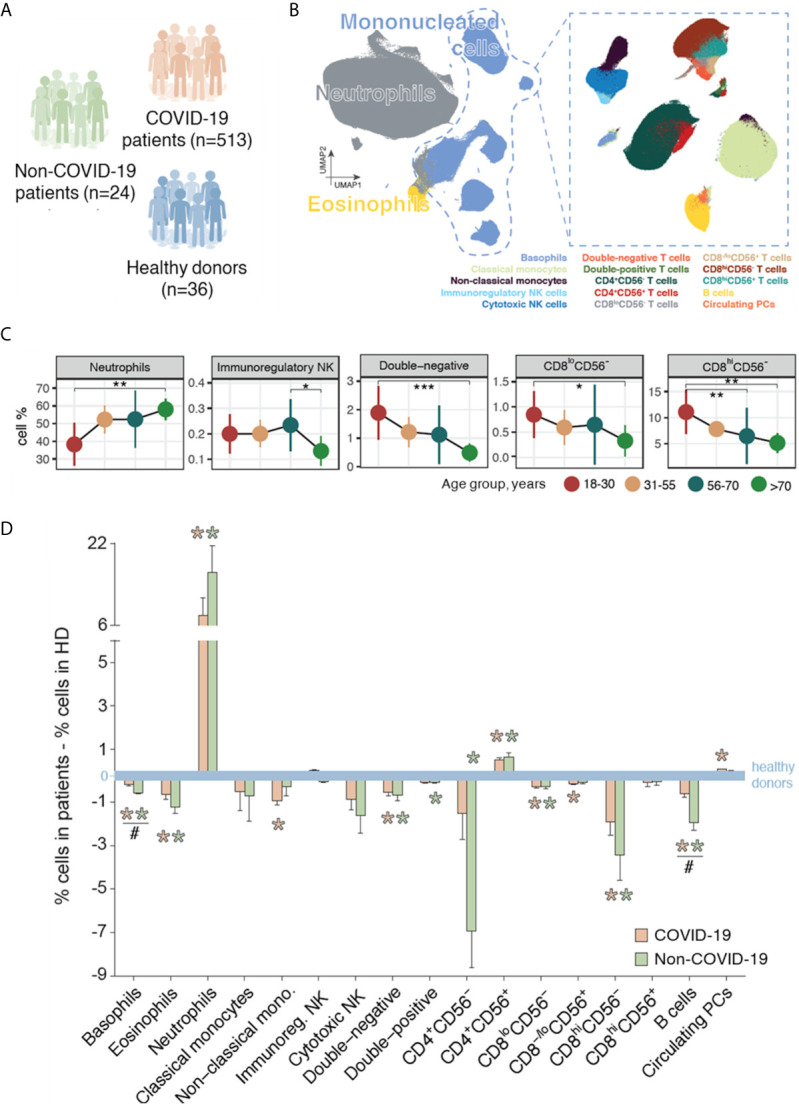
Immune profiling of patients with COVID-19, patients with non-SARS-CoV-2 infections, and healthy donors (HDs). **(A)** Immune profiling was performed using multidimensional flow cytometry in a training series of 513 patients with COVID-19, 24 patients with other infections, and 36 HDs. **(B)** Schematic representation of the 17 immune cell-types systematically identified through unbiased and semi-automated analysis in peripheral blood (PB) samples from all subjects included in the study (n=573). **(C)** Immune cell-type percentages in PB samples of HDs by age group (18–30 years, n=8; 31–55 years, n=8; 56–70 years, n=11; >70 years, n=9) for cell-types with significantly different levels across age groups. *P <0.05; **P <0.01; ***P <0.001 in all panels. Statistical significance was evaluated using the Kruskal–Wallis test, with multiple testing corrected using the Holm method. **(D)** Immune response in patients with COVID-19 (n=513) and in patients with other infections (n=24), illustrated as the variations in the median percentages of each cell-type *versus* the median values in age-group-matched HDs (n=36, blue line). Orange asterisks indicate significant differences between patients with COVID-19 and HDs, and green asterisks indicate significant differences between patients with other infections and HDs. Hash symbols (#) indicate significant differences between patients with COVID-19 and patients with other infections (*P* <0.05). Statistical significance was evaluated using Mann-Whitney test.

Data were analyzed using *FlowCT*, a semi-automated workflow we developed for deconvolution of immunophenotypic data and objective reporting on large datasets (**Figures 1A, B**) (32). Briefly, this four-step approach involves: 1) reading data; 2) building a self-organizing map using *FlowSOM* (version 1.14.1) (33) for clustering and dimensionality reduction; 3) building a minimum spanning tree to connect nodes according to their similarity; and 4) computing an automated meta-clustering by grouping similar nodes. The meta-clustering step is critical for the definition of cell populations; in this phase, groups of similar nodes are “fused” per specific algorithms [*ConsensusClusterPlus* (34) (version 1.46.0) R package] to obtain more consistent populations. *FlowCT* identified 17 immune cell-types for this analysis: basophils, eosinophils, neutrophils, classical and non-classical monocytes, immunoregulatory (CD16^-^CD56^hi^) and cytotoxic (CD16^+^CD56^lo^) NK cells, eight T cell subsets (double-negative, double-positive, CD4^+^CD56^-^, CD4^+^CD56^+^, CD8^lo^CD56^-^, CD8^-/lo^CD56^+^, CD8^hi^CD56^-^, CD8^hi^CD56^+^), B cells, and circulating plasma cells (PCs) (**Figure 1B**, **Supplemental Figure 1**, **Supplemental Table 3**).

### Fluorescence-Activated Cell Sorting

Various myeloid subsets and antigen-presenting cells from 11 COVID-19 patients and 4 HDs were stained with the combination HLADR-PacB, CD45-OC515, CD16-FITC, CD203c-PE, CD33-PerCPCy5.5, CD123-APC, CD14-APCH7 (**Supplemental Table 2**), and isolated in a MoFlo Astrios EQ sorter (Beckman Coulter, Brea, CA, USA). Based on its six-way sorting, basophils, myeloid and plasmacytoid dendritic cells (DC), classical and non-classical monocytes and neutrophils were simultaneously isolated from PB samples of 11 COVID-19 patients and 4 HDs with a purity greater than 95%. The gating strategy and mRNA expression of key markers that define each cell type are shown in **Supplemental Figure 2**. All cell types were successfully isolated in all cases except for plasmacytoid DCs and basophils in 2 patients. Cells were stored in Lysis/Binding Buffer (Invitrogen™, CA, USA).

### RNA-Sequencing (RNA-Seq) and Data Analysis

RNA-seq was performed using a protocol adapted from massively parallel single-cell RNA-sequencing (35), which enabled preparing libraries with as few cells as starting material. Briefly, we barcoded RNA from each sample in a retrotranscription (RT) reaction with AffinityScript Multiple Temperature Reverse Transcriptase (Agilent, Santa Clara, CA, USA) and different RT primers. After qPCR, cDNA with similar Ct values were pooled together. cDNA was purified with SPRIselect 1.2X (Beckman Coulter, Brea, CA, USA) and *in vitro-*transcribed with the T7 polymerase (New England Biolabs –NEB-, Ipswich, MA, USA) using the T7 promoter as template, introduced in the previous RT reaction. Samples were incubated for 16 hours at 37°C. RNA molecules were fragmented with 2 µL of 10X Zn^2+^ fragmentation buffer (Ambion™, ThermoFisher, Waltham, MA, USA) for 1 min at 70°C and purified with SPRIselect 2X. Afterwards, a ssRNA adaptor (Illumina, San Diego, CA, USA) was ligated to the 3’end of the RNA fragments in the presence of DMSO, 100 mM ATP, 50% PEG and T4 RNA ligase I (NEB, Ipswich, MA, USA) for 2 hours at 22°C. A second RT reaction was performed with AffinityScript Multiple Temperature Reverse Transcriptase and resulting cDNA was purified with SPRIselect 1.5X. Finally, cDNA was amplified with 12.5 µL Kappa Hifi ready mix + 1 µL of primer mix at 25 µM per sample and purified with SPRIselect 0.7X. Qubit, TapeStation and qPCR analyses were done as quality controls and the final library products at 4 nM were sequenced in a NextSeq 500 (Illumina, San Diego, CA, USA).

Raw sequencing data were demultiplexed through *bcl2fastq* software (version 2.20.0, Illumina, San Diego, CA, USA) and aligned to the GRCh38 human genome using *STAR* aligner (version 2.7.3a) (36). Matrix with gene counts were generated with *quant3p* [a wrapper based on *HTSeq* dynamics (37)]. Differential gene expression across all comparisons (COVID-19 patients *vs* HDs, and patients with favorable *vs* fatal outcome) of sorted immune populations was analyzed with *Deseq2* R package (version 1.28.1) (38). Functional enrichment analysis was performed through *ClusterProfiler* R package (39) considering an adjusted *P* < 0.05.

### Statistical Analysis

Immune profiles were compared between groups of patients/subjects using proportions or absolute levels of immune cell-types. The Kruskal–Wallis and Mann–Whitney tests were used to estimate the statistical significance observed between groups, and the χ^2^ test was used to test distributions between immunotypes resulting from unsupervised clustering. Multiple comparisons were corrected by the Holm method.

For multivariable analysis of baseline clinical and immune factors associated with overall survival (OS), a double Cox regression approach was performed: first, a univariable model was used to evaluate the prognostic value of each individual immune cell-type, as well as other clinical features, and then a multivariable regression was conducted using variables with *P*<0.05 and hazard ratio (HR)>5 on univariable analysis. Cutoff values for prognostic associations of individual immune cell-type percentages with COVID-19 outcome (alive *vs* dead) were those providing maximum sensitivity and specificity on receiver operating characteristic (ROC) curves. Survival probabilities were estimated using the Kaplan–Meier method; between-group differences were tested for statistical significance with two-sided log-rank tests, and HRs, plus two-sided 95% confidence intervals (CIs), were estimated using Cox regression models. Survival time was measured from initial PB sampling at presentation.

All statistical analyses were performed using the GraphPad Prism software (version 7, San Diego, CA, USA), SPSS (version 25.0.0, IBM, Chicago, IL, USA), and R (versions 3.5.1 and 4.0.0 for MFC and RNA-seq studies, respectively). *P* values of <0.05 were considered statistically significant.

## Results

### Patient Characteristics and Disposition

Among the 513 COVID-19 patients of the exploratory cohort, median age at presentation was 60 years (range, 19–94) and 49%/51% were male/female (**Table 1**). The most frequent comorbidities were hypertension (35%), diabetes (11%), cardiovascular disease (11%), and hypercholesterolemia (10%). Overall, 395 (77%) patients were hospitalized, including 32 (6%) who required ICU admission, and 23 (4%) died from COVID-19 (**Table 1**). At data cutoff, median follow-up in COVID-19 patients was 59 days.

**Table 1:** Demographics, comorbidities, and medical care received among COVID-19 patients, overall and according to disease outcome.

Characteristics	All (N = 513)	Survived (N = 490)	Died (N = 23)
**Age, median (range), years**	60 (19–94)	59 (19–94)	75 (31–93)
**Male, no. (%)**	253 (49%)	240 (49%)	13 (57%)
**Comorbidities, no (%)**			
Any	256 (50%)	236 (48%)	20 (87%)
Diabetes	58 (11%)	51 (10%)	7 (30%)
Hypercholesterolemia	52 (10%)	49 (10%)	3 (13%)
Hypertension	178 (35%)	163 (33%)	15 (65%)
Cardiovascular disease	56 (11%)	47 (10%)	9 (39%)
Solid tumor	15 (3%)	14 (3%)	1 (4%)
Hematological tumor	10 (2%)	7 (1%)	3 (13%)
**Medical care, no. (%)**			
Non-hospitalized	118 (23%)	118 (24%)	0 (0%)
Hospitalized	395 (77%)	372 (76%)	23 (100%)
ICU	32 (6%)	19 (4%)	13 (57%)
**Hospitalization, median (range), days**	8 (1–50)	7 (1–50)	12 (1–32)
**Follow-up, median (range), days**	59 (1–129)	63 (1–129)	12 (1–62)

ICU, intensive care unit.

### Immune Profiling of HDs

HDs aged >70 years had a significantly higher percentage of neutrophils and significantly lower percentages of immunoregulatory NK cells *versus* younger age groups (**Figure 1C**). Percentages of double-negative, CD8^lo^CD56^-^, and CD8^hi^CD56^-^ T cells progressively reduced with increasing age. Percentages of other immune cell-types by age group are shown in **Supplemental Figure 3**; there was a non-significant trend (*P*=0.11) towards a lower B-cell percentage with increasing age.

### Immune Response in COVID-19 Patients

At presentation, COVID-19 patients (n=513) showed significant alterations in the proportions of 11 of the 17 immune cell-types investigated, compared to age-matched HDs (**Figure 1D**). The median percentage of neutrophils was 8% higher (*P*=0.014), whereas there were significant reductions in the median percentages of basophils, eosinophils, and non-classical monocytes. There were also significant reductions in the median percentages of four T cell subsets (double-negative, CD8^lo^CD56^-^, CD8^–/lo^CD56^+^, CD8^hi^CD56^-^) but a significant increase in CD4^+^CD56^+^ T cells; similarly, there was a significant increase in the percentage of mature circulating PCs and a significant reduction of B cells. The immune profile of the COVID-19 patients was generally similar to that of the 24 patients with non-SARS-CoV-2 infections in terms of differences *versus* age-matched HDs, except for significantly lower reductions in the percentages of basophils and B cells (**Figure 1D**). Collectively, these results suggest that most COVID-19 patients have an immune profile in PB consistent with an active immune response.

### Activation and Differentiation of Innate and Adaptive Immune Cells After SARS-CoV-2 Infection

**Figure 2:**
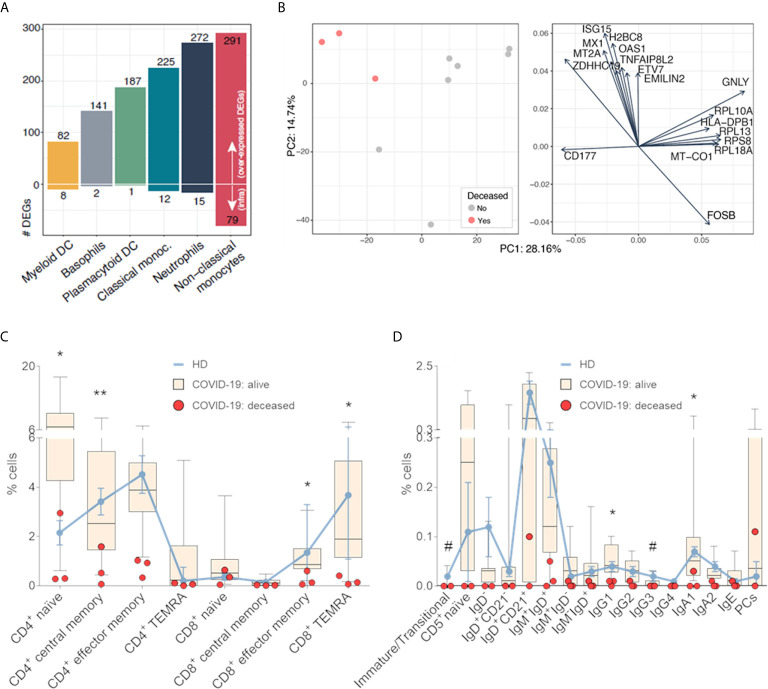
Activation and differentiation of innate and adaptive immune cells after SARS-CoV-2 infection. **(A)** Number of differentially expressed genes (DEGs) in myeloid dendritic cells (DC), basophils, plasmacytoid DC, classical monocytes, neutrophils and non-classical monocytes, isolated from peripheral blood (PB) samples of COVID-19 patients (n=11) and age-matched healthy donors (HDs, n=4). The number of under- and over-expressed DEGs is depicted, and cell types were ordered from the lowest to the highest number of DEGs. **(B)** Principal component analysis of RNA-seq data from neutrophils showing partial segregation between COVID-19 patients with favorable *vs* fatal outcome (left panel). DEGs in neutrophils from COVID-19 patients as shown in left panel (right panel). Percentage of antigen-dependent differentiation of **(C)** T and **(D)** B cell subsets in the PB of 14 COVID-19 patients (11 alive and 3 deceased) and 4 age-matched HDs. Lines represent median values in HDs; boxes correspond to minimum-to-maximum values in alive patients; and dots indicate individual deceased patients. *P <0.05; **P <0.01 between alive and deceased COVID-19 patients. Hash symbol (#) indicates significant differences between HDs and COVID-19 patients (*P* <0.05). Statistical significance was calculated using the Mann-Whitney test. CM, central memory; EM, effector memory; TEMRA, effector memory re-expressing CD45RA T cells.

We performed RNA-seq in various myeloid and DC subsets isolated by FACS, to further investigate immune cell activation in COVID-19 patients (n=11) *vs.* HDs (n=4). There was a progressive increment of differentially expressed genes (DEGs) from myeloid DC into basophils, plasmacytoid DC, classical monocytes, neutrophils and non-classical monocytes (**Figure 2A**). Most DEGs were over-expressed, with overlapping functional enrichment across different cell types related to antimicrobial humoral response, inflammatory response regulation and neutrophil activation (**Supplemental Figure 4A**). The low number of under-expressed genes in myeloid DC was significantly associated with adaptive immune response regulation, including B cell proliferation and immunoglobulin production (**Supplemental Figure 4B**). A complete list of all DEGs per cell type is provided in the **Supplemental Table 4**. Of note, principal component analysis of RNA-seq data on neutrophils from COVID-19 patients, showed partial segregation between those with favorable *vs* fatal outcome (**Figure 2B**). Genes involved in antiviral activity (i.e., *ISG15*, *MX1*, *OAS1*) and hyper-responsiveness of the immune system (i.e., *TNFAIP8L2*) were over-expressed in neutrophils from deceased COVID-19 patients (**Figure 2B**).

We also performed more comprehensive immunophenotyping of the T and B cell compartments to characterize antigen-dependent differentiation of both cell types after SARS-CoV-2 infection (n=14). Based on reference values from HDs (n=4), there were no significant differences in the distribution of CD4 and CD8 T cell naïve and memory subsets (**Figure 2C**). Similarly, the relative distribution of 14/16 subsets within the B cell compartment was similar between COVID-19 patients and HDs, except for the percentage of immature/transitional and IgG3 memory B cells (**Figure 2D**). However, there was a general trend for lower percentages of multiple T and B cell subsets in PB of patients with fatal (n=3) *vs* favorable (n=11) outcome. Statistical significance in CD4 naïve and effector memory T cells, CD8 effector memory and effector memory re-expressing CD45RA (TEMRA) T cells was observed (**Figure 2C**), as well as IgG1 and IgA1 memory B cells (**Figure 2D**). Taken together, the transcriptional and immunophenotypic data suggest an association between COVID-19 severity and neutrophil activation as well as reduced levels of adaptive immune subsets. These findings urged further analysis in the larger cohort to unequivocally identify poor outcome associated immune signatures.

### Immunotype Identification and Association With Patient Characteristics and Clinical Outcome

Lymphopenia and neutrophilia have been associated with increased risk of severe COVID-19 (2, 40, 41). Analysis of absolute cell-type numbers among COVID-19 patients (n=513) showed significant associations between lower lymphocyte (*P* <0.001) and higher neutrophil (*P*=0.001) counts and fatal COVID-19 (**Supplemental Figure 5**). However, there was substantial heterogeneity in absolute leukocyte numbers among the 23 COVID-19 patients who died; eight (35%) had lower, four (17%) similar, and 11 (48%) higher leukocyte counts than the median value in HDs. These data provide the rationale for using cell-type percentages rather than absolute numbers for subsequent analyses to provide more meaningful results.

**Figure 3:**
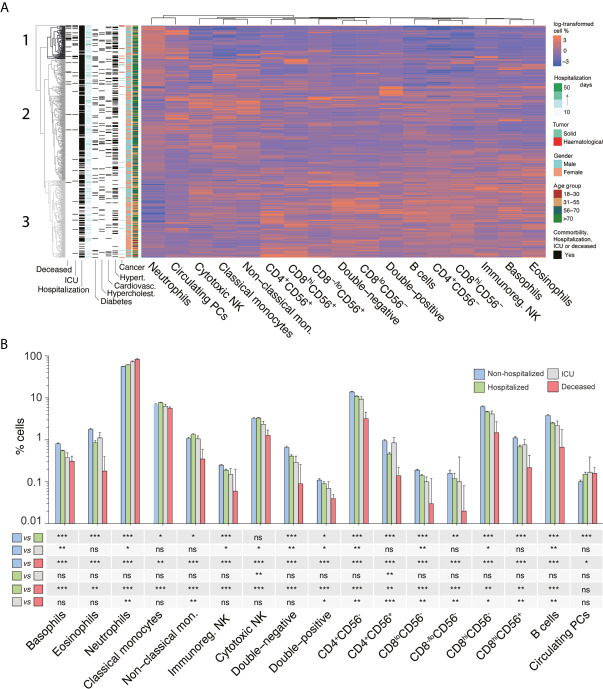
The immune landscape of patients with COVID-19 and its association with disease severity. **(A)** Unsupervised clustering of 513 patients with COVID-19 based on the relative distribution of 17 immune cell types in peripheral blood (PB) samples taken at presentation. For the columns to the left of the cell-percentage data, moving from right to left, patient rows are color-coded according to age and gender; green and red marks indicate the patients with solid or hematological tumors; dark gray marks indicate the presence of the comorbidities of hypertension, cardiovascular disease, hypercholesterolemia, and diabetes; light blue-to-green marks indicate duration of hospitalization; and dark gray marks indicate patients requiring hospitalization (n=395), patients who needed intensive care unit (ICU) admission (n=32), and patients who died (n=23). **(B)** Median percentages of the 17 immune cell-types in PB samples from patients with COVID-19 who were not hospitalized (n=118) and those who required hospitalization (n=395), as well as the subsets of hospitalized patients who required ICU admission (n=32) and/or who died from COVID-19 (n=23). *P <0.05; **P <0.01; ***P <0.001; ns, not significant. Statistical significance was evaluated using Mann–Whitney tests.

Unsupervised clustering analysis according to the relative distribution of the 17 immune cell-types identified three distinct immunotypes among the 513 COVID-19 patients (**Figure 3A**). The first branch of the analysis segregated a single patient (0.2%) with diffuse large B-cell lymphoma and therapy-related severe neutropenia who died from COVID-19 shortly after admission. The second branch separated a group of 74 (14.4%) patients (immunotype 1, dark gray) from the remaining 438 (85.4%) patients. These patients were subsequently divided at the third branch into two subgroups containing 268 (52.3%; immunotype 2, gray) and 170 (33.1%; immunotype 3, light gray) patients, respectively.

Patients with immunotype 1 were characterized by significantly (*P*<0.0001) lower percentages of all immune cell-types except for neutrophils and circulating PCs, which were increased, compared to patients with immunotypes 2 and 3 (**Supplemental Figure 6**). Immunotype 3 was characterized by an opposite distribution pattern to immunotype 1, and immunotype 2 had intermediate features between immunotypes 1 and 3. There were also significant differences in age distributions, gender proportions, rates of comorbidities, medical care required, median hospitalization duration, and incidence of fatal COVID-19 between the three immunotypes (**Table 2**).

**Table 2:** Demographics and clinical characteristics of COVID-19 patients (n=513) clustered according to their immune cell composition during SARS-CoV-2 infection.

Characteristics	Unclustered patient (N = 1; 0.2%)	Immunotype 1 (N = 74; 14.4%)	Immunotype 2 (N = 268; 52.3%)	Immunotype 3 (N = 170; 33.1%)	Significance
**Age (years)**					
18–30	0 (0%)	1 (1%)	2 (0.7%)	16 (9%)	b,c
31–55	1 (100%)	24 (32%)	76 (28%)	88 (52%)	a,b
56–70	0 (0%)	22 (30%)	104 (39%)	32 (19%)	b,c
>70	0 (0%)	27 (36%)	86 (32%)	34 (20%)	a,c
**Female, no. (%)**	1 (100%)	31 (42%)	121 (45%)	107 (63%)	b,c
**Comorbidities, no. (%)**					
Any	1 (100%)	49 (66%)	145 (54%)	61 (36%)	b,c
Diabetes	0 (0%)	13 (18%)	33 (12%)	12 (7%)	ns
Hypercholesterolemia	0 (0%)	7 (9%)	30 (11%)	15 (9%)	ns
Hypertension	0 (0%)	33 (45%)	109 (41%)	36 (21%)	b,c
Cardiovascular disease	0 (0%)	14 (19%)	32 (12%)	10 (6%)	b
Cancer	1 (100%)	7 (9%)	11 (4%)	6 (4%)	ns
**Medical care, no. (%)**					
Non-hospitalized	0 (0%)	4 (5%)	41 (15%)	73 (43%)	a,b,c
Hospitalized	1 (100%)	70 (95%)	227 (85%)	97 (57%)	a,b,c
ICU	1 (100%)	16 (22%)	9 (3%)	6 (4%)	a,b
**Hospitalization, median, days**	26	10	7	8	a,c
**Outcome, no. (%)**					
Died	1 (100%)	14 (19%)	7 (3%)	1 (0.6%)	a,b

ICU, intensive care unit.

a, significant difference between immunotypes 1 and 2.

b, significant difference between immunotypes 1 and 3.

c, significant difference between immunotypes 2 and 3.

ns, no significant differences among immunotypes.

In accordance with these findings by immunotype, percentages of neutrophils and circulating PCs were progressively higher and percentages of all other immune cell-types progressively lower when comparing non-hospitalized patients *versus* hospitalized patients *versus* patients requiring ICU admission *versus* patients who died (**Figure 3B**). Overall, these results illustrate that the relative distribution of all immune cell-types in PB at presentation is significantly associated with specific pre-existing medical conditions and clinical outcome in COVID-19 patients.

### Clinical and Immune Prognostic Factors for Survival: Multivariable Analysis

Optimal cutoffs for association with survival were determined for percentages of each of the 17 immune cell-types based on ROC curves; using these cutoffs, percentages of all immune cell-types except circulating PCs predicted significantly different overall survival (OS) (**Supplemental Figure 7A**). Similar results were obtained using absolute cell numbers (**Supplemental Figure 7B**). The increased risk of death was greatest in COVID-19 patients with <1% *versus* ≥1% B cells (HR 17.1 [95% CI 7.0–41.6], *P *<0.0001) and ≥76.22% *versus* <76.22% neutrophils (HR 14.9 [95% CI 5.9–37.7], *P *<0.0001). Clinical prognostic factors associated with significantly poorer OS were age >70 years (*P*=0.0002) and presence of any pre-existing medical conditions (*P*=0.004) (data not shown).

**Figure 4:**
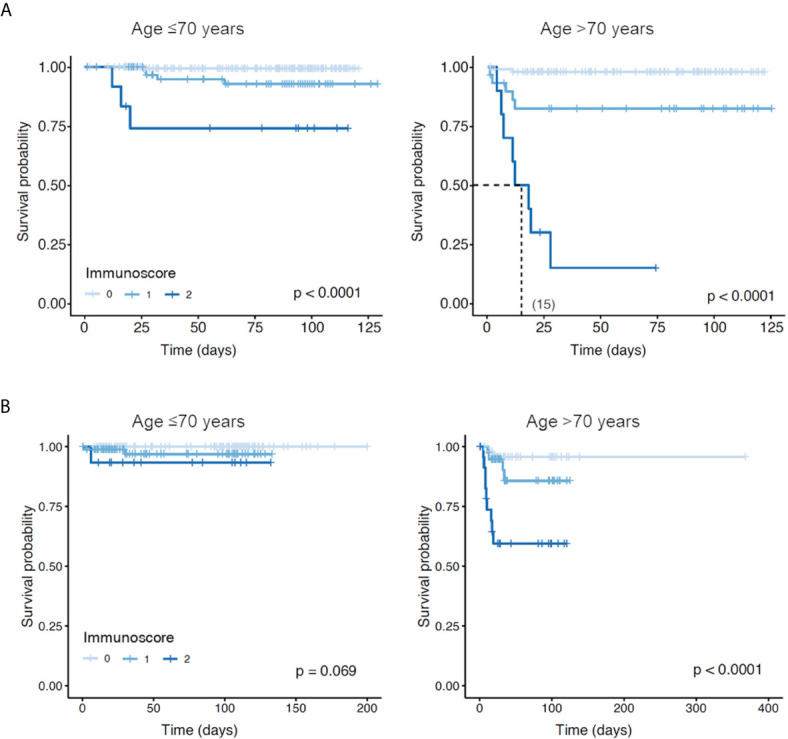
Overall survival of patients with COVID-19 according to presence of two high-risk immune biomarkers identified in this study. **(A)** Patients had an immunoscore of 0, 1, or 2 according to the absence or the presence of one or two risk factors, respectively: <0.67% non-classical monocytes and <1% B cells. Among patients aged ≤70 years, 274, 80, and 12 had an immunoscore of 0, 1, or 2, respectively. Among patients aged >70 years, 107, 30, and 10 had an immunoscore of 0, 1, or 2, respectively. **(B)** This immunoscore was further validated in an independent cohort of additional 355 patients with COVID-19, where 126, 91, and 15 patients aged ≤70 years had an immunoscore of 0, 1, or 2, respectively; whereas 55, 43, and 25 patients aged >70 years had an immunoscore of 0, 1, or 2, respectively.

On multivariable analysis, which included the 11 immune cell-types with significant prognostic value and HR above 5, plus age and presence of comorbidities, the relative percentages of non-classical monocytes (HR 4.2 [95% CI 1.2–14.1], *P*=0.02) and B cells (HR 4.1 [95% CI 1.2–13.7], *P*=0.02), and age >70 years (HR 4.0 [95% CI 1.6–10.2], *P*=0.003) were identified as independent prognostic factors for OS (**Supplemental Table 5**). This immunoscore comprising two immune cell-type risk-factors (<0.67% non-classical monocytes; <1% B cells) was significantly predictive of fatal COVID-19 based on ROC analysis (area under the curve 0.86 [95% CI 0.77–0.95], *P *<0.001; **Supplemental Figure 8**), and stratified COVID-19 patients (n=513) aged either ≤70 years or >70 years into groups with significantly different OS (**Figure 4A**). OS rates at 1 month were 99%, 97%, and 74% in patients aged ≤70 years and 98%, 82.5%, and 15% in patients aged >70 years who had 0, 1, or 2 risk-factors (*P *<0.0001), respectively. The median OS for patients aged >70 years with an immunoscore of two was 15 days. In the 355 patients of the validation series, OS rates at 1 month were 100%, 97%, and 93% in patients aged ≤70 years and 96%, 95%, and 60% in patients aged >70 years who had 0, 1, or 2 risk-factors, respectively (**Figure 4B**). These results thus identify two high-risk immune biomarkers that are independent of age and pre-existing medical conditions.

### Divergent Immune Response Trajectories According to COVID-19 Outcome

**Figure 5:**
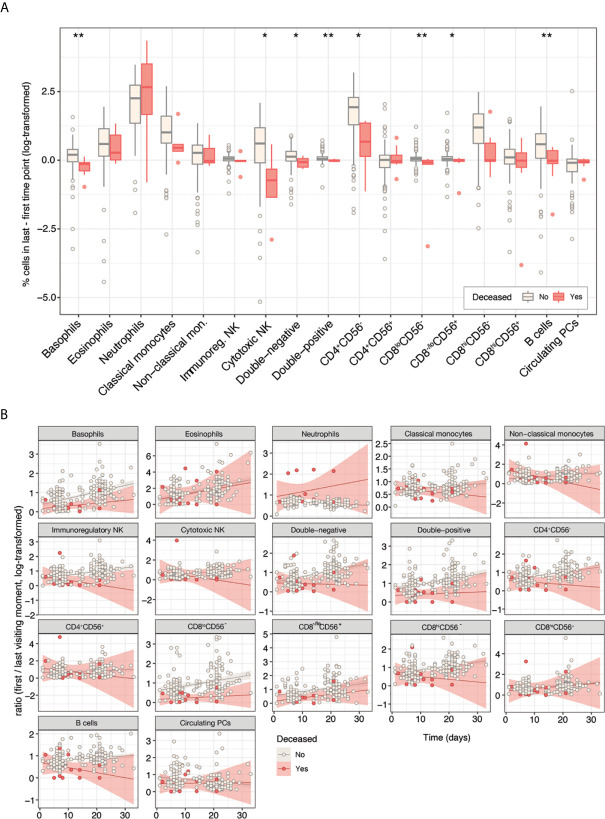
Divergent immune response trajectories in patients with COVID-19 with longitudinal immune monitoring who had favorable (n=158) or fatal (n=9) outcomes. **(A)** Absolute variations in percentages of immune cell-types from first to last PB sampling time point, according to outcome. *P <0.05; **P <0.01. Statistical significance was evaluated using the Kruskal–Wallis test. **(B)** Longitudinal relative variations in percentages of immune cell-types from first through all subsequent PB sampling time points, according to outcome.

Among the 167 COVID-19 patients with immune monitoring during follow-up, there were significantly different changes in the relative distribution of eight immune cell types from first to last PB sample between patients who survived (n=158) *versus* those who died (n=9). The latter showed reductions in percentages of basophils and cytotoxic NK cells over time, together with static percentages of double-negative, double-positive, CD8^lo^CD56^-^, and CD8^-/lo^CD56^+^ T cells, and B cells. CD4^+^CD56^-^ T cells increased in patients who died, but not by as much as in patients who survived (**Figure 5A**).

Longitudinal immune monitoring confirmed the divergent immune trajectories between patients who survived or died, as demonstrated in immune cell-type subsets including basophils, CD8^lo^CD56^-^ T cells, and B cells (**Figure 5B**). These data on immune profile at presentation and during follow-up demonstrate that the level of B cells is of importance throughout the disease course, whereas levels of specific innate and adaptive cell-type subsets emerge as particularly relevant during hospitalization.

## Discussion

Severe COVID-19 occurs more frequently in the elderly and in those with comorbidities, leukocytosis, or lymphopenia (4, 42). However, the relationships between age or clinical history and immune dysfunction, as well as between disease severity and immune response over time, remain poorly understood (1, 40, 43–45). Indeed, most of the initial studies were performed in small series of patients and compared immune profiles in patients with moderate or severe disease, or in those who recovered from COVID-19. In the present study, we conducted a holistic and unbiased analysis of the immune cell phenotype during SARS-CoV-2 infection in a large cohort of 513 patients who reflected the wide age-range and medical history associated with COVID-19. Our findings provide new insight into disease immunopathology and identify key immune cell-types that are significantly associated with OS, regardless of age and comorbidities.

Immune profiles have previously been compared between COVID-19 patients and HDs in relatively small numbers of subjects and without age-matching (11, 16, 21, 22). We showed that the relative distribution of immune cell-types in PB is affected by aging; these changes are characterized by an expansion of neutrophils together with progressively reduced levels of certain NK and T cell subsets. Our findings highlight that comparisons of immune profiles between COVID-19 patients and HDs should be age-matched. Consistent with previous studies (22), we observed higher percentages of neutrophils as well as transcriptional activation of this plus other myeloid subsets and DCs in COVID-19 patients *versus* HDs. Conversely, we have not observed significant downregulation of HLA-DR and other activation markers in antigen-presenting cells in COVID-19 patients compared with healthy adults. However, it should be noted that differences in gene expression may emerge according to the anatomical location of cells (46) and disease severity. Indeed, reduced HLA-DR expression appears to be more pronounced in patients with severe COVID-19 infection (16, 18, 47–50). Thus, the small number of patients analyzed herein with RNA-seq may have limited the identification of deregulated antigen presentation. Nevertheless, when considered together with other studies showing elevated percentages of circulating PCs (10, 11, 16, 21, 22), our data suggest that most COVID-19 patients display an expansion, activation, and differentiation of multiple immune lineages during SARS-CoV-2 infection. These findings are consistent with the neutralization of infection and the asymptomatic or mild-to-moderate course of the disease observed in 90% of cases (2, 4).

It remains unclear whether severe COVID-19, which is observed in 5–10% of patients (2, 4), arises due to the characteristics of the viral infection, patient demographics and comorbidities, depth of immune response, or all three. In the present study, we reveal the presence of an immunotype in 14% of patients that is characterized by an abnormally greater expansion of neutrophils and circulating PCs, together with a profound reduction of all other immune cell-types, and associated with fatal outcome. Such changes could be surrogates for both innate and adaptive over-reactivity to SARS-CoV-2 infection, with heightened inflammation potentially leading to the depletion of other effector cells (10). These results confirm previous observations in smaller series that showed increased neutrophil levels and reduced percentages of multiple cell-types in patients with severe COVID-19 (10, 11, 16, 21, 22, 40), and unequivocally support a role for the host immune response in determining the disease course.

Humoral immunity is critical for viral clearance and SARS-CoV-2 elicits a robust B cell response, as evidenced by the rapid and near-universal detection of virus-specific neutralizing antibodies (51). Notwithstanding, most studies investigating prognostic immune biomarkers for disease severity have focused on total lymphocyte counts, the neutrophil-to-lymphocyte ratio, or the neutrophil-to-CD8-T-cell ratio (1, 4, 20, 22, 43, 52). We found that, among all leukocyte subsets with prognostic value, the percentage of B cells at presentation showed the strongest association with OS; patients with <1% B cells had a 17-fold greater risk of death *versus* those with ≥1% B cells. To our knowledge, this is one of the first studies supporting a prominent role for B cells in determining COVID-19 severity; in this context, it might be hypothesized that plasma or neutralizing antibodies from recovered patients (53–55) could be used as immunoprophylaxis for patients with a low percentage of B cells (51, 56). A percentage of neutrophils of >76% was also strongly associated with inferior OS in our study. An exacerbated inflammatory response associated with SARS-CoV-2 infection could cause enhanced neutropoiesis and increased neutrophil influx into the PB, in accordance with recent observations of immature neutrophils in the PB of patients with severe COVID-19 (16, 18, 24). Thus, a predominant differentiation of hematopoietic stem cells into the neutrophil lineage, reduced numbers of lymphoid progenitors in elderly individuals’ bone marrow (57), as well as elevated cytokine levels (58), could all contribute to the reduction of virtually all lymphocyte subsets in the PB of patients with severe COVID-19. Accordingly, we also showed for the first time that the percentage of immature/transitional B cells (which egress from the bone marrow into PB) was significantly lower in COVID-19 patients *vs* HDs.

Additionally, we identified the percentage of non-classical monocytes as an independent prognostic factor in the multivariable analysis of OS. These data confirm recent findings reported in 86 COVID-19 patients, in which a decreased percentage of non-classical monocytes in PB discriminated between moderate/severe and mild disease (24). Lower levels of non-classical monocytes could be due to their activation (as suggested by our RNA-seq data) and migration from the PB into the inflamed lungs of critically ill patients; accordingly, proinflammatory monocyte-derived macrophages have been found to be abundant in the bronchoalveolar lavage fluid from patients with severe COVID-19 (12). An important aspect of our work is that, by combining these two independent immune prognostic factors – percentages of B cells and non-classical monocytes – we were able to develop an immunoscore for risk-stratification that resulted in significant OS differences among patients aged ≤70 years and those aged >70 years. These results are clinically relevant because severe COVID-19 is unusual in younger patients who do not have pre-existing medical conditions, and presence of these high-risk immune biomarkers could help physicians tailor their management of these patients (4). Although our immunoscore did not reach statistical significance in the prediction of OS in younger patients of the validation cohort, this could be explained by their better clinical management during the subsequent outbreaks of COVID-19, which are not being as unpredictable as the first one. Nevertheless, we confirmed the usefulness of the immunoscore in older patients in both series. Then, these biomarkers might indicate the need for innovative treatment strategies to overcome otherwise unsurmountable severe COVID-19.

It has been speculated that critical illness caused by SARS-CoV-2 infection can be a result of an immune response that is too weak, leading to virus-induced pathology, or too strong, leading to immune-induced pathology (21). We hypothesized that longitudinal immune monitoring could shed light on these differing immune response trajectories and help understand the variability in clinical outcome that is not indicated solely by the immune response at presentation. Our results build upon initial observations suggesting that patients with severe COVID-19 had progressive lymphopenia together with increasing neutrophil counts in PB (1, 20, 43). Our data also reveal divergent immune response trajectories with respective to other innate and adaptive immune cell-types between COVID-19 patients with favorable *versus* fatal outcomes.

Overall, considering our findings in HDs and in COVID-19 patients at presentation and during follow-up, it could be hypothesized that an overly mature immune status could affect a patient’s ability to produce an immediately effective humoral response. Consequently, sustained inflammation due to prolonged viral load would further compromise the feasibility of neutralizing SARS-CoV-2 due to progressively lower levels of NK and T cells. Thus, our study may contribute to a better understanding of disease immunopathobiology and provides a simple tool for risk-stratification of COVID-19 patients based on immune status at presentation. This immunoscore is readily applicable and may be widely utilized as, unfortunately, the number of new confirmed cases of COVID-19 remains high (https://covid19.who.int/).

## Data Availability Statement

The datasets generated in this study can be found in the GEO database (https://www.ncbi.nlm.nih.gov/geo/) and are available with the accession numbers GSE153610 and GSE155897.

## Ethics Statement

The studies involving human participants were reviewed and approved by Clinica Universidad de Navarra Ethics Committee. The patients/participants provided their written informed consent to participate in this study.

## Author Contributions

BP and JY conceived the idea and designed the study protocol. SI, AL-D, CP, LB, and DA performed flow cytometry immunophenotyping. EM-S, CMa, SI, AL-D, CP, AZ, CMo, and BP analyzed flow cytometry data. SS and AV-Z performed experiments. FC-T, MM-O, FA, EM, MF-A, AA, MF-L, MR, TP-W, IP, MS, JA and JY provided study material and/or patients. EM-S, JG, CMa, CP, CB and BP performed statistical analysis. EM-S, JG, CMa, BP and JY wrote the manuscript. All authors contributed to the article and approved the submitted version.

## Funding

This work was supported by the Centro de Investigación Biomédica en Red – Área de Oncología - del Instituto de Salud Carlos III (CIBERONC; CB16/12/00369 and CB16/12/00489), Instituto de Salud Carlos III/Subdirección General de Investigación Sanitaria (FIS No. PI17/01243), Fondo Europeo de Desarrollo Regional (FEDER), Fundación BBVA, Departamento de Salud de Gobierno de Navarra (0011-3638-2020-000004), and Asociación Española Contra el Cáncer (FCAECC, Predoctoral Grant Junta Provincial Navarra). This study was supported internationally by Cancer Research UK, FCAECC and AIRC under the Accelerator Award Programme, and the European Research Council (ERC) 2015 Starting Grant (MYELOMANEXT).

## Conflict of Interest

The authors declare that the research was conducted in the absence of any commercial or financial relationships that could be construed as a potential conflict of interest.
