# Supplementary material for: Immunological Biomarkers of Fatal COVID-19: A Study of 868 Patients
Source: Front Immunol. 2021 May 3;12:659018. doi: 10.3389/fimmu.2021.659018 (PMC8126711; doi:10.3389/fimmu.2021.659018)
Supplement: Supplementary file 1 [file DataSheet_1.zip › Data Sheet 1 .docx]

Supplementary Material

**Supplemental Table 1.** Numbers of COVID-19 patients with one or more peripheral blood samples obtained at presentation (sample 1) and during the disease course.

| **COVID-19 patients, n** | | | **Samples obtained, n** | **Median time from first to last PB sample (days)** | | |
| --- | --- | --- | --- | --- | --- | --- |
| **All** | **Survived** | **Died** |  | **All** | **Survived** | **Died** |
| 513 | 490 | 23 | 1 | NA | NA | NA |
| 167 | 158 | 9 | 2 | 14 | 15 | 7 |
| 31 | 28 | 3 | 3 | 20 | 20 | 11 |
| 5 | 4 | 1 | 4 | 14 | 17 | 14 |
| 3 | 2 | 1 | 5 | 26 | 27 | 21 |
| 1 | 1 | 0 | 6 | 33 | 33 | NA |

NA, not applicable; PB, peripheral blood.

**Supplemental Table 2.** Description of the antibodies used in this study.

| **Panel** | **Antigen** | **Label** | **Clone** | **Cat #** | **Company** |
| --- | --- | --- | --- | --- | --- |
| Immuno-  phenotyping | CD3 | BV450 | UCHT1 | 560365 | BD |
|  | CD45 | BV500 | HI30 | 560777 | BD |
|  | CD20 | FITC | L27 | 345792 | BD |
|  | CD16 | PE | 3G8 | 555407 | BD |
|  | CD4 | PerCPCy5.5 | SK3 | 332772 | BD |
|  | CD19 | PECy7 | J3-119 | IM3628 | Beckman Coulter |
|  | CD56 | APC | NCAM16.2 | 341027 | BD |
|  | CD8 | APCH7 | SK1 | 641400 | BD |
| Cell sorting | HLADR | PacBlue | L243 | 307633 | Biolegend |
|  | CD45 | PacOrange | GA90 | CYT-450C | Cytognos |
|  | CD16 | FITC | 3G8 | 555406 | BD |
|  | CD203c | PE | 97A6 | IM3575 | Beckman Coulter |
|  | CD33 | PerCPCy5.5 | P67.6 | 333146 | BD |
|  | CD123 | APC | AC145 | 130-090-901 | Miltenyi Biotec |
|  | CD14 | APCH7 | MϕP9 | 641394 | BD |

BD, Beckton Dickinson Biosciences

**Supplemental Table 3.** Immunophenotypic profile of the 17 immune cell-types identified by automated clustering using *FlowCT*.

| **Cell type** | **Immunophenotype** |
| --- | --- |
| Basophils | SSC^lo^CD3^-^CD4^-^CD8^-^CD16^-^CD19^-^CD20^-^CD45^lo^CD56^-^ |
| Eosinophils | SSC^hi^CD3^-^CD4^-^CD8^-^CD16^-^CD19^-^CD20^-^CD45^+^CD56^-^ |
| Neutrophils | SSC^hi^CD3^-^CD4^-^CD8^-^CD16^+^CD19^-^CD20^-^CD45^lo^CD56^-^ |
| Classical monocytes | SSC^int^CD3^-^CD4^-^CD8^-^CD16^-^CD19^-^CD20^-^CD45^+^CD56^-^ |
| Non-classical monocytes | SSC^int^CD3^-^CD4^-^CD8^-^CD16^+^CD19^-^CD20^-^CD45^+^CD56^-^ |
| Immunoregulatory NK cells | SSC^lo^CD3^-^CD4^-^CD8^-^CD16^-^CD19^-^CD20^-^CD45^+^CD56^hi^ |
| Cytotoxic NK cells | SSC^lo^CD3^-^CD4^-^CD8^-^CD16^+^CD19^-^CD20^-^CD45^+^CD56^lo^ |
| Double-negative T cells | SSC^lo^CD3^+^CD4^-^CD8^-^CD16^-^CD19^-^CD20^-^CD45^+^CD56^-^ |
| Double-positive T cells | SSC^lo^CD3^+^CD4^+^CD8^+^CD16^-^CD19^-^CD20^-^CD45^+^CD56^-^ |
| CD4^+^CD56^-^ T cells | SSC^lo^CD3^+^CD4^+^CD8^-^CD16^-^CD19^-^CD20^-^CD45^+^CD56^-^ |
| CD4^+^CD56^+^ T cells | SSC^lo^CD3^+^CD4^+^CD8^-^CD16^-^CD19^-^CD20^-^CD45^+^CD56^+^ |
| CD8^lo^CD56^-^ T cells | SSC^lo^CD3^+^CD4^-^CD8^lo^CD16^-^CD19^-^CD20^-^CD45^+^CD56^-^ |
| CD8^-/lo^CD56^+^ T cells | SSC^lo^CD3^+^CD4^-^CD8^-/lo^CD16^-^CD19^-^CD20^-^CD45^+^CD56^+^ |
| CD8^hi^CD56^-^ T cells | SSC^lo^CD3^+^CD4^-^CD8^hi^CD16^-^CD19^-^CD20^-^CD45^+^CD56^-^ |
| CD8^hi^CD56^+^ T cells | SSC^lo^CD3^+^CD4^-^CD8^hi^CD16^-^CD19^-^CD20^-^CD45^+^CD56^+^ |
| B cells | SSC^lo^CD3^-^CD4^-^CD8^-^CD16^-^CD19^+^CD20^+^CD45^+^CD56^-^ |
| Circulating PCs | SSC^lo^CD3^-^CD4^-^CD8^-^CD16^-^CD19^lo^CD20^-^CD45^+^CD56^-^ |

NK, natural killer; PCs, plasma cells.

**Supplemental Table 4.** List of differentially expressed genes in 6 myeloid subsets from COVID-19 patients vs healthy donors.

**Supplemental Table 5.** Multivariable analysis of prognostic factors for overall survival.

| **Prognostic factors** | **HR** | **95% CI** | ***P* value** | |
| --- | --- | --- | --- | --- |
| Age (≤70 vs >70 years) | 4.0 | 1.6–10.2 | 0.003 |  |
| Percentage of non-classical monocytes (<0.67% vs ≥0.67%) | 4.2 | 1.2–14.1 | 0.02 |  |
| B cells (<1.0% vs ≥1.0%) | 4.1 | 1.2–13.7 | 0.02 |  |

CI, confidence interval; HR, hazard ratio for risk of death.


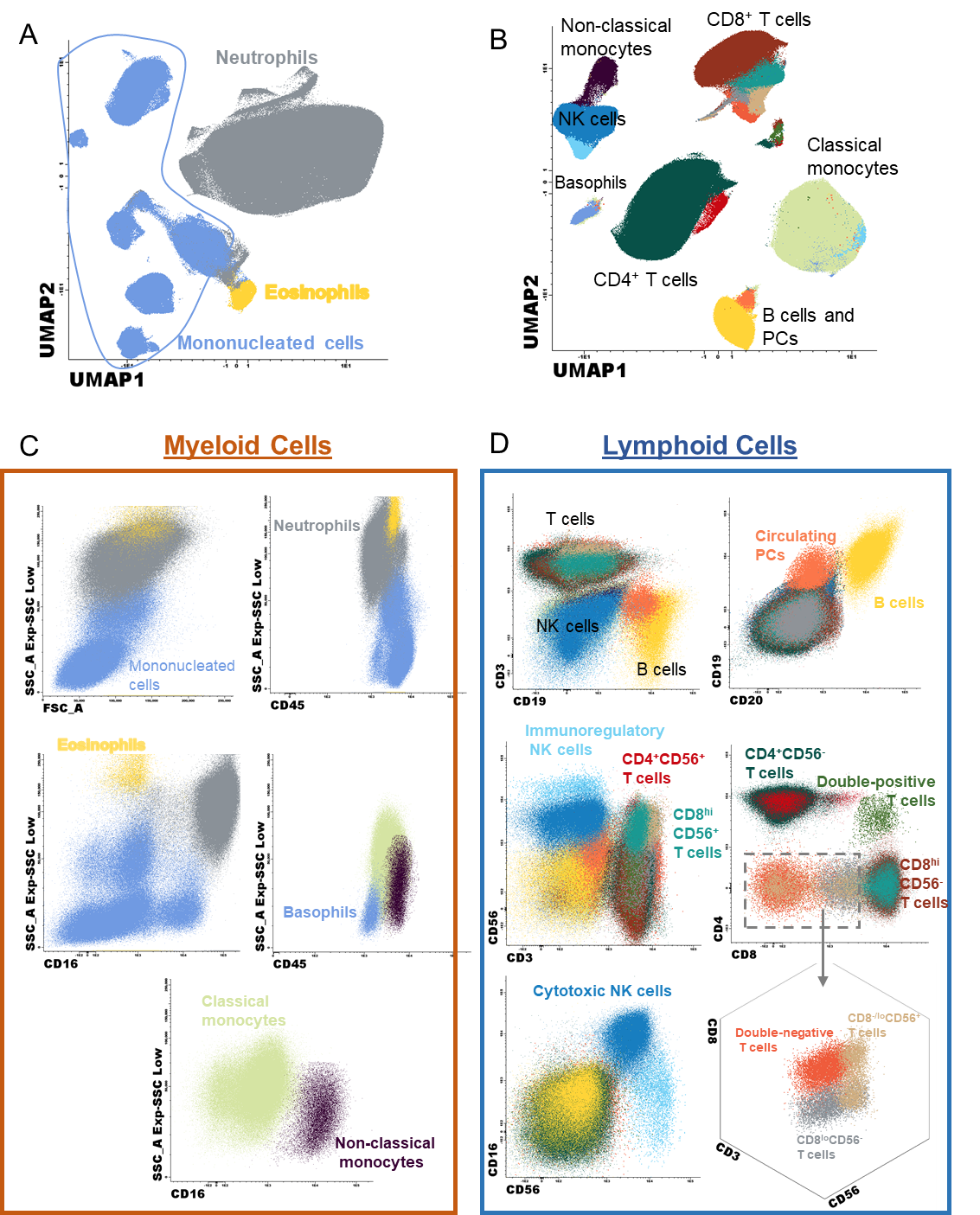


**Supplementary Figure 1.** Immunophenotypic profile of the 17 immune cell-types identified by automated clustering using *FlowCT*. **(A)** Dimensional reduction by Uniform Manifold Approximation and Projection (UMAP) representing polynucleated (neutrophils and eosinophils) and mononucleated cells. **(B)** Subclustering of mononucleated cells by UMAP. **(C)** Identification of myeloid cell types: eosinophils (yellow), neutrophils (grey), basophils (blue), classical monocytes (light green), and non-classical (purple) monocytes. **(D)** Identification of lymphoid cell subsets: NK cells (CD3^-^CD19^-^CD56^+^), T cells (CD3^+^CD19^-^CD56^-^), and B cells (CD3^-^CD56^-^CD19^+^). NK cells were further divided into immunoregulatory (CD56^high^CD16^-/dim^, light blue) and cytotoxic (CD56^dim^CD16^high^, dark blue) subsets. Based on CD3, CD4, CD8, and CD56 expression, multiple T cell subsets were defined: CD4^+^CD56^-^ (dark green), CD4^+^CD56^+^ (red), CD4^+^CD8^+^ double-positive (green), CD8^+^CD56^-^ (brown), CD8^+^CD56^+^ (turquoise green), CD4^-^CD8^-^ double-negative (dark orange), CD8^lo^CD56^-^ (light grey), and CD8^-/lo^CD56^+^ (light brown). B cells (CD19^+^CD20^+^, yellow) and circulating plasma cells (PCs; CD19^lo^CD20^-^, light orange) were characterized by the expression of CD19 and CD20.


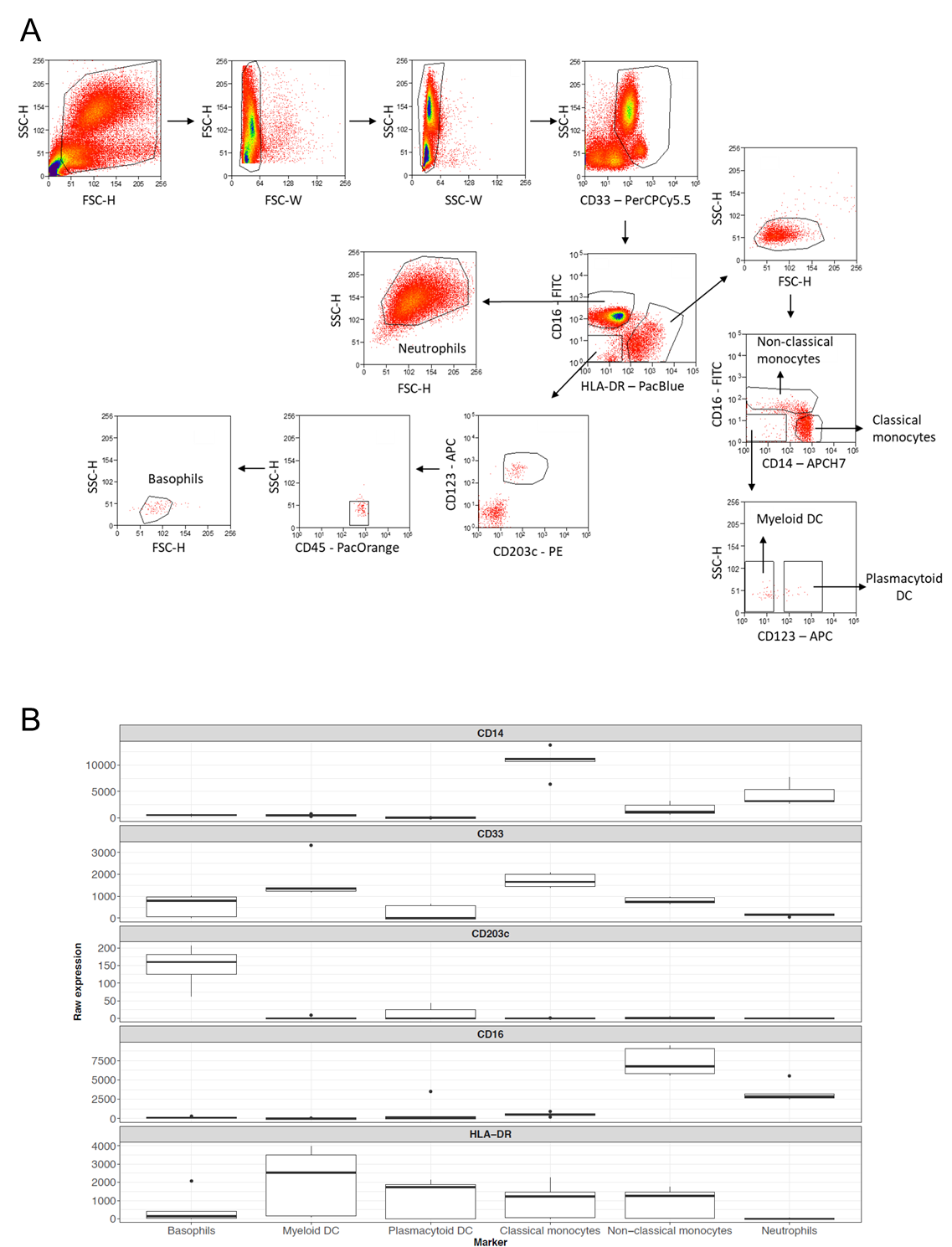


**Supplemental Figure 2. FACS sorting of myeloid subsets. (A)** Gating strategy for the isolation of 6 myeloid cell types: neutrophils (SSC^hi^CD33^+^CD16^+^HLA-DR^-^), non-classical monocytes (SSC^int^CD33^+^HLA-DR^+^CD16^+^CD14^-^), classical monocytes (SSC^int^CD33^+^HLA-DR^+^CD16^-^CD14^+^), myeloid dendritic cells (DC) (SSC^int^CD33^+^HLA-DR^+^CD16^-^CD14^-^CD123^-^), plasmacytoid DC (SSC^int^CD33^+^HLA-DR^+^CD16^-^CD14^-^CD123^+^), and basophils (SSC^lo^CD33^+^HLA-DR^-^CD123^+^ CD203c^+^). **(B)** Purity of sorted cells was assessed by measuring the mRNA expression levels of selected genes that are key in each myeloid cell subset, such as *CD14*, *CD33*, *ENPP3* (CD203c), *FCGR3A* (CD16), and *HLA-DRA* (HLA-DR).


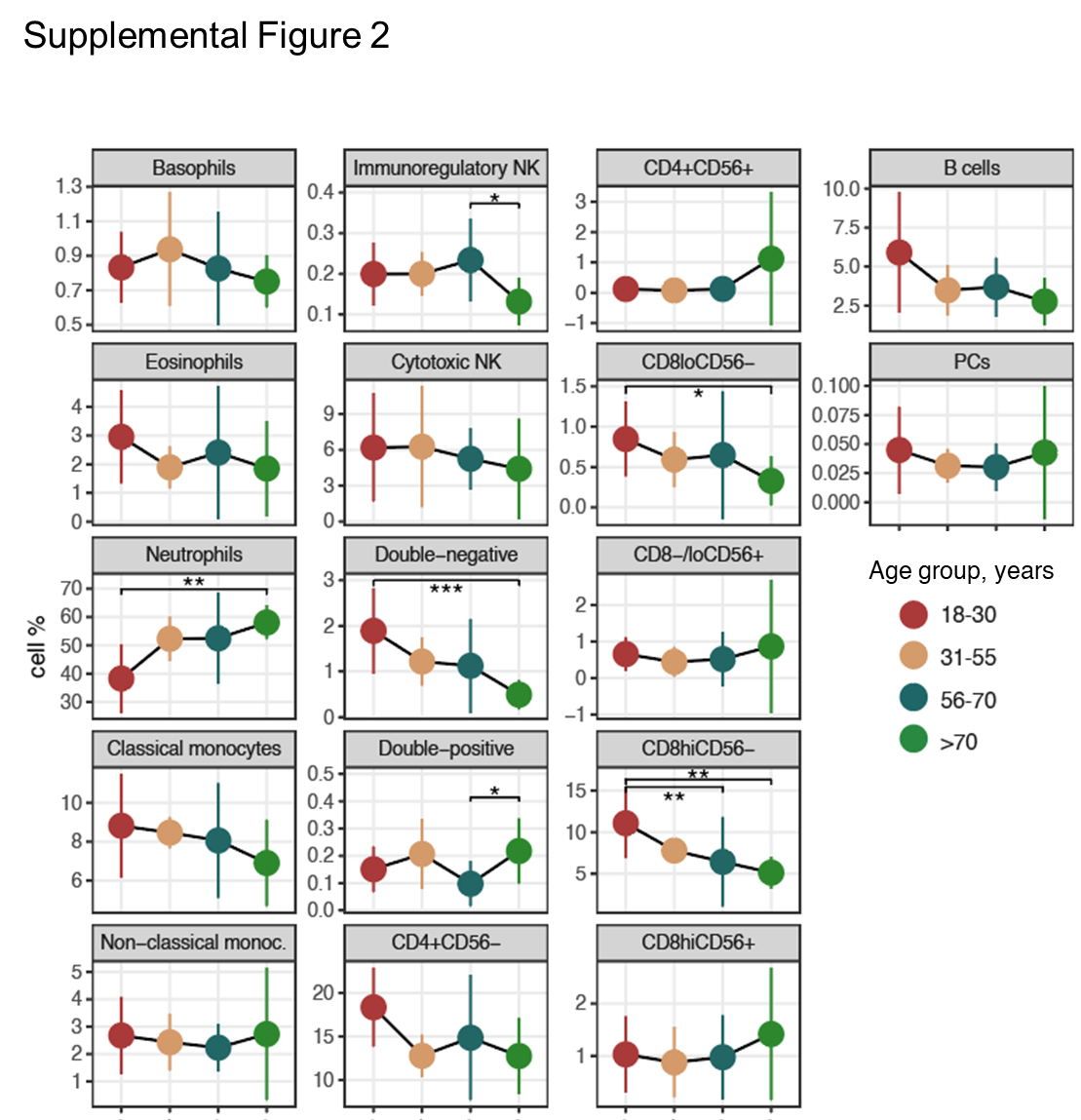


**Supplemental Figure 3.** Percentages of the 17 immune cell-types in the peripheral blood of healthy donors by age group: 18–30 years (n=8), 31–55 years (n=8), 56–70 years (n=11), and >70 years (n=9). *, *P* < 0.05; **, *P* < 0.01; ***, *P* < 0.001.


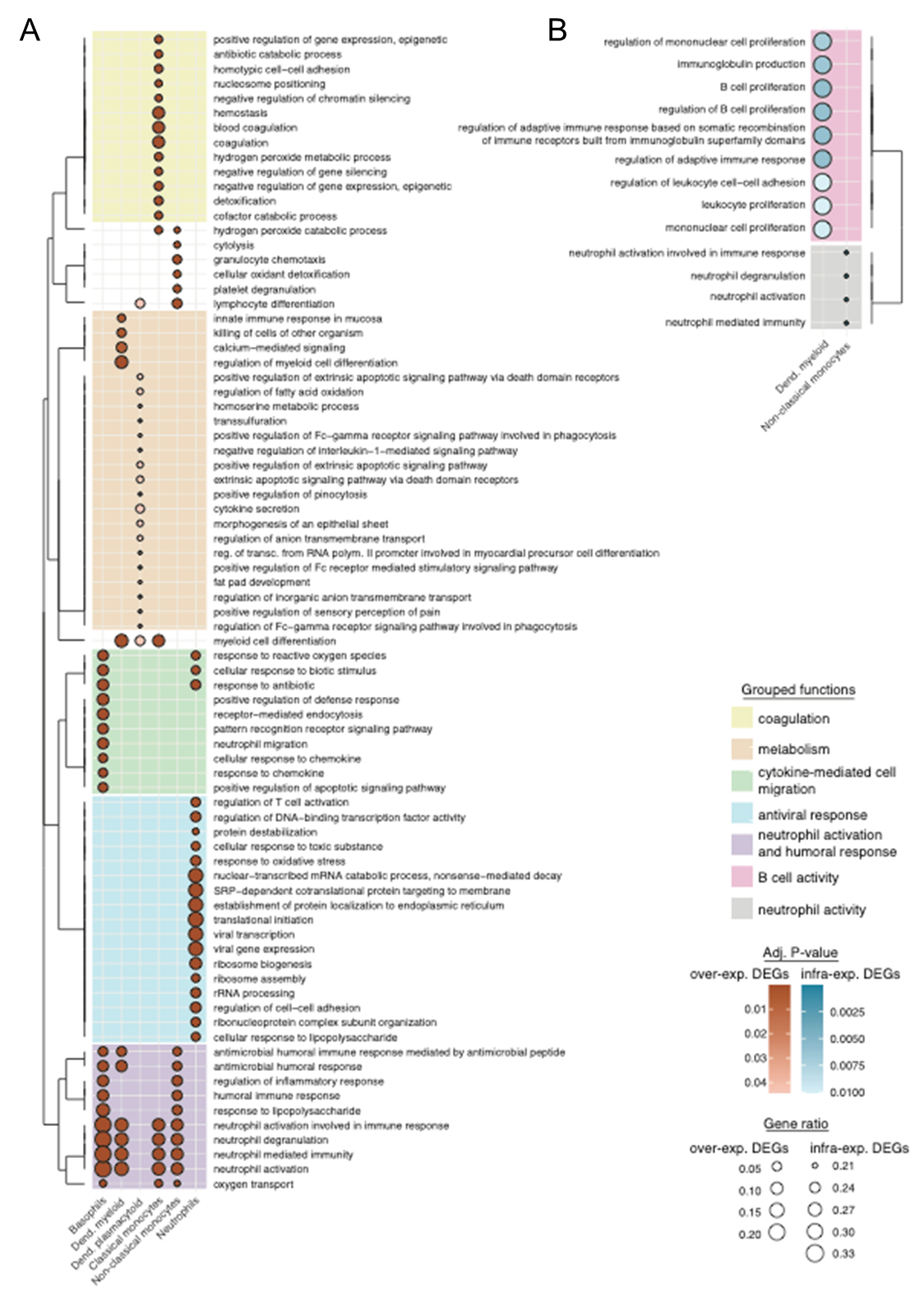


**Supplemental Figure 4. Cellular pathways altered in myeloid cells in COVID-19 patients compared to healthy donors.** Significant Gene Ontology (GO) sets based on genes over-expressed **(A)** and infra-expressed **(B)** in cell types isolated from peripheral blood of COVID-19 patients (n=11) *vs* age-matched healthy donors (n=4). The color intensity of each GO correlates with the adjusted *P*-value logarithmic transformed (the more significant, the darker the color). The size of each dot indicates the gene ratio for each GO (i.e., the number of differentially expressed genes inside the function’s gene dataset).


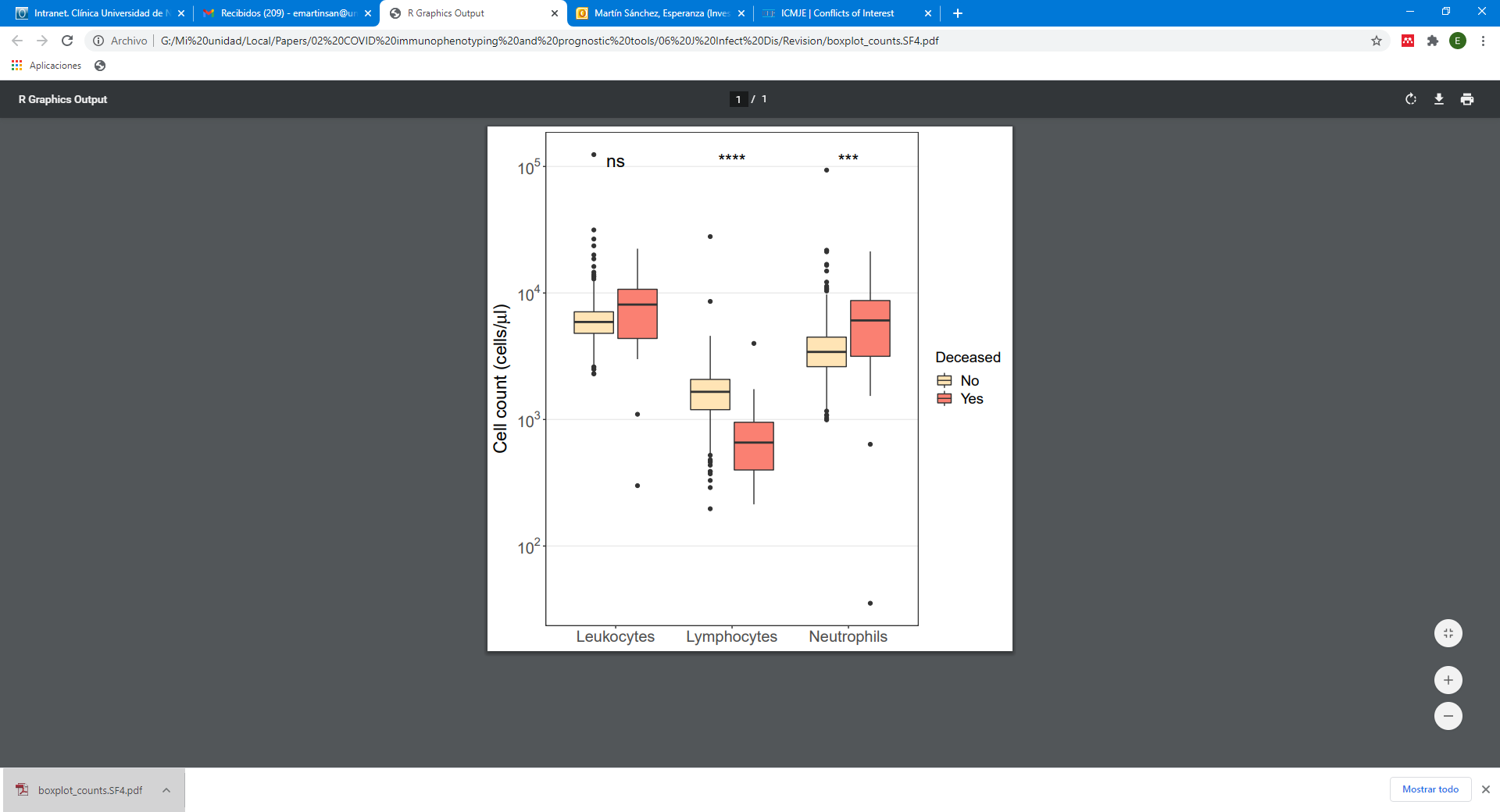


**Supplemental Figure 5.** Absolute numbers of leukocytes, lymphocytes, and neutrophils in the peripheral blood of patients with COVID-19 at presentation, according to outcome (orange boxes, surviving patients, n=490; red boxes, patients who died, n=23). ***, *P* < 0.001; ****, *P* < 0.0001; ns, not significant.


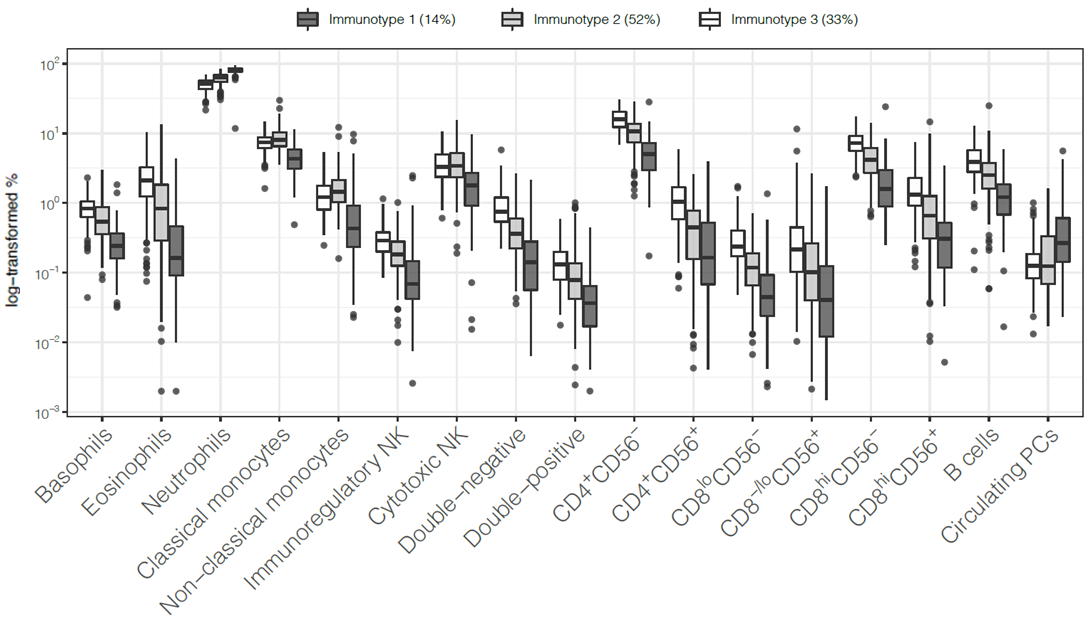


**Supplemental Figure 6.** Relative distributions of the 17 immune cell-types in peripheral blood samples from patients with COVID-19 at presentation, according to immunotype (see Figure 2A). *P* < 0.0001 for all comparisons across immunotypes.

**
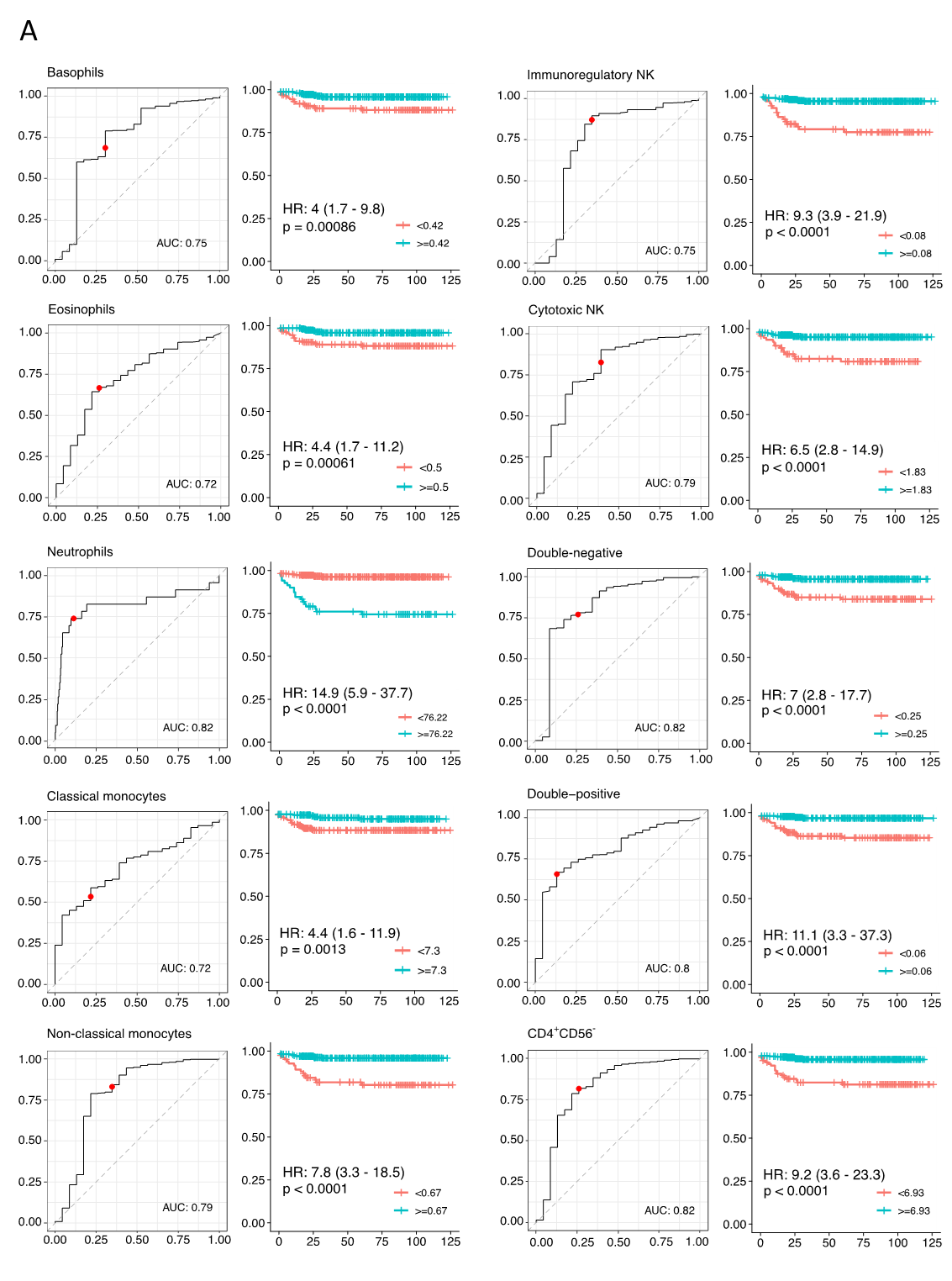
**

**
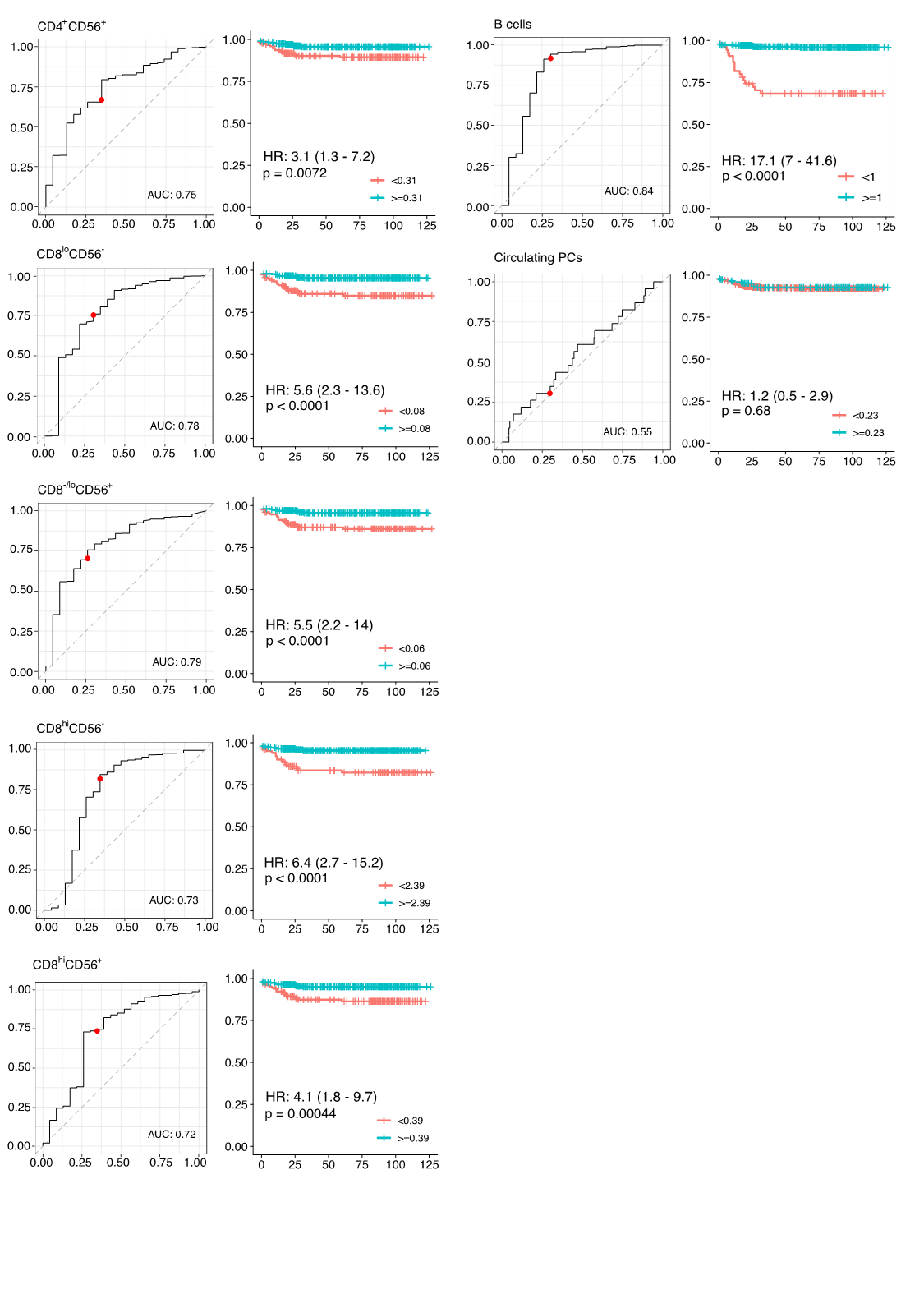
**

**
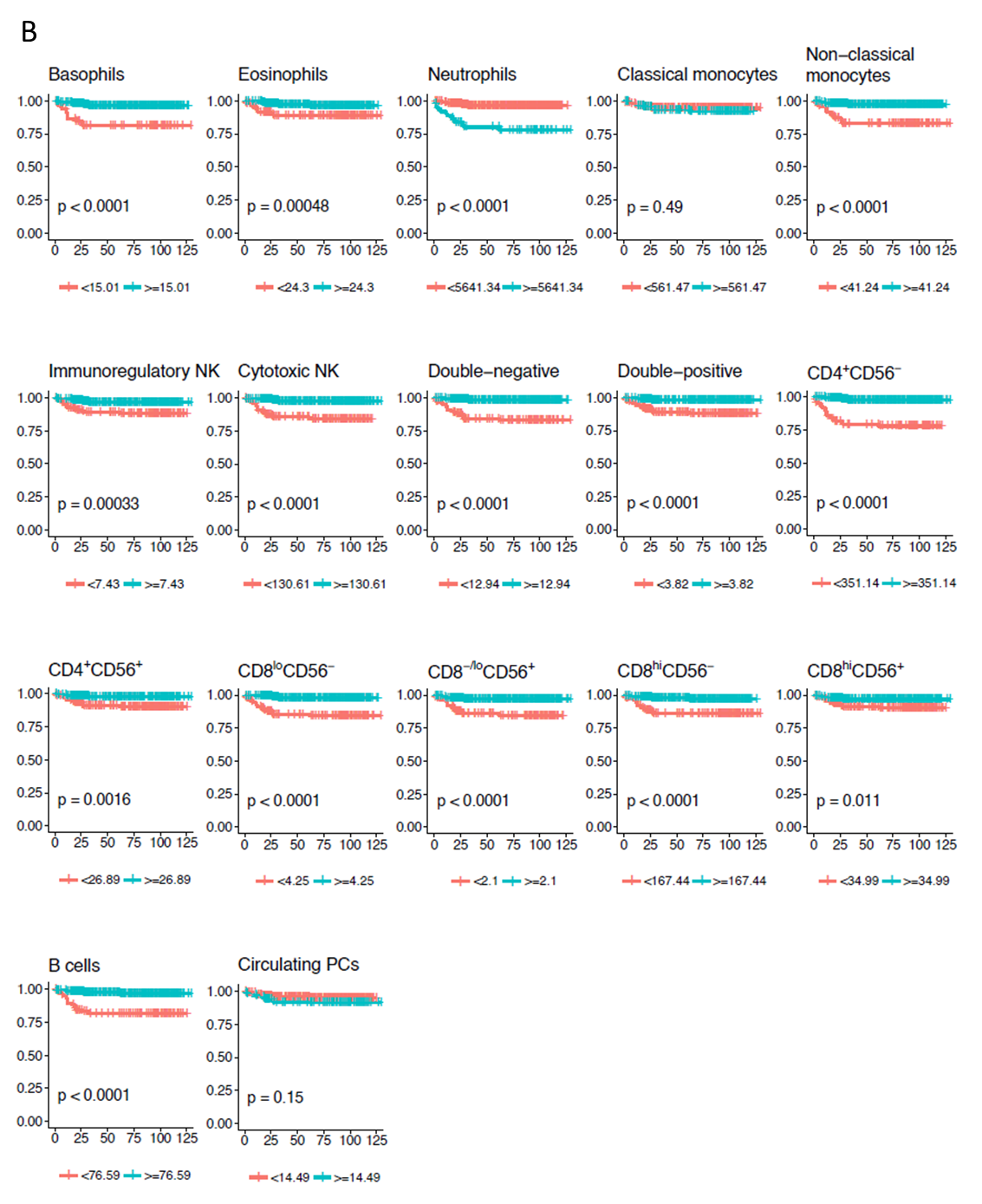
**

**Supplemental Figure 7.** Receiver operating characteristic (ROC) curves of predictive specificity and sensitivity for survival, and Kaplan–Meier distributions of overall survival (OS) according to the **(A)** percentages of each immune cell-type in the peripheral blood of patients with COVID-19 at presentation (n=513). Red dots in the ROC curves show cutoff values yielding maximum specificity and sensitivity; these cutoffs are indicated in the legends of the respective Kaplan–Meier curves and were used to define patient subgroups for OS analysis. Hazard ratios (HR) and statistical significance of differences in OS between groups were determined by Cox regression univariate analysis. On the Kaplan–Meier curves, time (*x*-axis) is shown in days. Analyses were also conducted according to the **(B)** absolute numbers of each immune cell-type; only the Kaplan–Meier curves and the p-values are shown for these analyses.

**
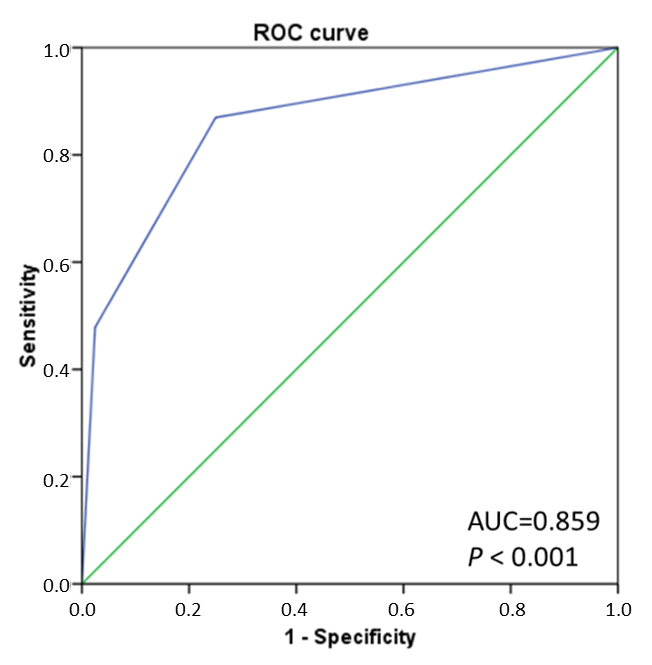
**

**Supplemental Figure 8.** Receiver operating characteristic (ROC) analysis of the sensitivity and specificity of an immunoscore for predicting patients who died from COVID-19. The immunoscore (0, 1, 2) comprises two risk factors: <0.67% non-classical monocytes and <1% B cells. AUC: area under the curve
